# Primary and Secondary Fracture Dressings

**Published:** 1885-06

**Authors:** J. McF. Gaston

**Affiliations:** Professor of the Principles and Practice of Surgery, Atlanta, Ga.


### PRIMARY AND SECONDARY FRACTURE DRESSINGS.

A CLINICAL LECTURE, DELIVERED AT THE SOUTHERN MEDICAL COLLEGE, BY J. MCF. GASTON, M. D., PROFESSOR OF THE PRINCIPLES AND PRACTICE OF SURGERY, ATLANTA, GA.

Reported for THE ATLANTA MEDICAL AND SURGICAL JOURNAL.

Gentlemen: The case before you illustrates the different modes of treating fractures of the leg with temporary and permanent appliances. You will recollect that a double fracture of the tibia with a fracture of the fibula occurred in this case from the falling of a heavy piece of timber across the limb. After extension and counter-extension with co-aptation of the fragments, a roller bandage was applied from the toes of the foot to the knee, and two light board splints, extending from the knee below the foot, being well padded, were adjusted to the inner and outer aspect of the limb by the turns of a roller with a long compress laid upon the spine of the tibia. I drew your attention on the occasion to the importance of having the breadth of the splints to correspond with the slope of the leg, so that a uniform support should be given anteriorly and posteriorly by the roller, and that additional padding should be placed around the maleoli to prevent the pressure of the splints upon these projections. As was seen at the end of a week, there was no undue inflammation in any part, and only slight ecchymosed patches near the seat of fracture midway the leg, while the apposition of the fragments was accurate, so as to preserve the proper form of the limb. It was determined then to replace the board splints by the plaster of Paris dressing, and a modification of it was made by drawing two ordinary woolen stockings of different numbers over the limb before applying the crinoline saturated with plaster. As you observed, this adaptation of the foot and leg of the lady's stocking to every part serves better than the leg of a pair of flannel drawers which has been applied for a similar purpose, and by using two layers of the material, the setting of the plaster upon the outer stocking does not affect the pliancy and softness of that within, which is next the skin.

But now, fifteen days since this plaster dressing was applied, it is found that from the subsidence of the slight swelling which existed, and perhaps some shrinking of the tissues from the constant elevation of the limb in the swing support, there is not that fixidity that is requisite to secure the best result in uniting the bone. Instead of breaking up the moulding of this cast, a line along the front of an inch in width has been cut away, so that by pressure upon the sides of the sheath there is some cracking of the plaster upon the posterior part, and the edges are brought together, giving a firm support to every part of the leg. It is observed now that, with strips of cloth around the ankle and at the middle and upper part of the leg outside of the plaster moulding, it is secured so as to adapt itself to the limb, and can be graduated as occasion may require. When the patient is lying with the leg suspended, the strips of bandage can be slackened, and when he rises or moves about on his crutches, which may be done with safety, they may be tightened, thus affording the most favorable conditions for the restoration of the bony structure. This affords a good modification of the Bavarian splint, which consists in two layers of flannel united together by a seam on the posterior part of the leg, and sewing that within, while that outside is slightly stitched around the limb, so that after applying the plaster, the latter can be released and the two sides can be opened or closed at will. This dressing is well suited to compound fractures, where the plaster splint is desirable for more uniform support, and especially in multiple fractures.

There is a question among surgeons as to the propriety of using a primary plaster dressing for fractures, and all the doubtful features are reconciled by adopting such a moulding as the above, since the pressure can be graduated according to the degree of swelling.

The use of stocking material may subserve our purposes admirably in adapting the angle made by the foot upon the leg, after opening a line with the scissors in front, to injuries near the elbow or the shoulder. It is seen how nicely the turn fits the elbow, with the leg of the stocking extending up the posterior part of the arm and the opened foot reaching down upon the forearm. And again you perceive that when the foot of the stocking covers the shoulder with the leg extending down the arm, that the acromian process is accommodated within the angle of the heel. Fractures near the upper or lower articulations of the humerus call for angular splints, and by adopting the stocking material, soaked with the silicate of soda solution, a splint may be moulded accurately to the desired form, and it hardens promptly, while it is much lighter than the plaster of Paris.

---

WHAT IS DENTAL SURGERY ?—We note that in nearly all of the States and in the District of Columbia laws are being enacted limiting the practice of "Dental Surgery" to a certain privileged class which is designated as above. Now, if it is intended that physicians shall not treat any disease of the mouth unless they take out a degree from some dental "college," or from some medical school where there is "annually delivered a full course of lectures and instructions in dentistry or dental surgery," as the Kansas Dental Law has it, it is about time that medical men were looking to their interests. The above law, however, generously permits "physicians, surgeons, or others," to extract teeth! If the dental "profession" has any claim to consider itself a specialty in medicine, this course is calculated to exclude it utterly. What would be thought if ophthalmologists, otologists or gynecologists were to secure legislative enactment to prevent their medical colleagues from practicing in their field unless they adopted the "profession" of ophthalmology, otology, or gynecology, by taking out a "degree" in these respective branches? Such laws must be unconstitutional, or at least in conflict with the charters of medical schools, which are supposed to send out graduates empowered to treat any of the ills to which the flesh is heir.—*N. Y. Med. Record.*

